# Formation Mechanism and Motion Characteristics of Multiple Jets in Spherical Section Free Surface Electrospinning

**DOI:** 10.3390/ma18040908

**Published:** 2025-02-19

**Authors:** Jing Yin, Lan Xu

**Affiliations:** National Engineering Laboratory for Modern Silk, College of Textile and Clothing Engineering, Soochow University, Suzhou 215123, China; yinjing@alu.suda.edu.cn

**Keywords:** spherical section free surface electrospinning, multiple jets, jet motion, numerical simulation

## Abstract

In this study, during the efficient preparation of nanofibers using a spherical section free surface electrospinning (SSFSE) device with different sphere radii, the formation mechanism and motion characteristics of multiple jets were thoroughly investigated through the numerical simulation method. The mechanical model of multiple jets was established, and the key role of electric field intensity in the formation and motion of jets was defined; in addition, the relationship between the jet initial velocity and the electric field intensity distribution on the solution surface was established. On this basis, a magnetohydrodynamic model was introduced, and a turbulence model as well as a volume of fluid model were combined to numerically simulate the jet motion during the SSFSE process. The results showed that as the sphere radius increased, the maximum velocity of the jets gradually decreased. However, the area of multiple jets generated increased, and the interaction force between the jets increased, resulting in a more obvious outward expansion of the jet trajectory. Therefore, the optimal SSFSE device with a sphere radius of 75 mm was determined. Finally, the results of numerical simulation were verified by experiments using a polymeric solution with low conductivity. This study can play a guiding role in effectively increasing the number of jets per unit area of solution surface in actual production, thus achieving continuous, uniform, and efficient preparation of micro-/nanofibers.

## 1. Introduction

Micro-/nanofibers have a large specific surface area, a high aspect ratio, porosity, and active sites, which are widely used in biomedicine [1], packaging [2], sensors [3], energy [4], and other fields. Electrospinning is one of the simplest methods to prepare continuous micro-/nanofibers. By adjusting solution properties, spinning parameters, and environmental conditions, micro-/nanofibers with controllable diameter, structure, and morphology can be prepared [5,6]. However, with the traditional single-needle electrospinning technique it is easy to clog the needle, and the technique can only produce a single jet in the spinning process, which limits its application in industrialization [7,8]. Therefore, various needleless electrospinning techniques, such as roller electrospinning [9], splashing electrospinning [10], bubble electrospinning [11,12], centrifugal electrospinning [13], and free surface electrospinning [14,15,16,17,18,19], have been developed to increase the number of Talyor cones and improve the yield of micro-/nanofibers. In the initial studies of the free surface electrospinning (FSE) technique, Yarin et al. [20] mixed permanent magnet suspensions into the spinning solution to form a normal magnetic field for disturbing the fluctuations of the solution surface. This method was limited to polymers that could be mixed well with oil-based magnet suspensions. Through the electric field force without the assistance of other mechanical external forces, the subsequently developed FSE techniques can generate multiple jets by stimulating the solution surface [14,21]. By designing the structures and sizes of the spinneret, the electric field distribution during the spinning process can be optimized, resulting in higher quality and a high yield of nanofibers [12,15,19].

In recent years, some theoretical studies have been carried out to completely understand the mechanisms which affect the formation and movement of jets in needleless electrospinning techniques. The electric field intensity and solution properties are key factors affecting the jet generation, as well as the formation and quality of nanofibers [14,22,23]. Based on the dispersion law, Lukas et al. [24] revealed a strong correlation between the critical electric field intensity and the number of jets for FSE, which unveiled the effects of spinneret structures and solution properties on the jets. According to the electrohydrodynamic instability, Qin et al. [14,25] studied the influence of surface tension and electric field intensity on the interjet distance in the stepped-pyramidal spinneret spinning process and found that high-yield nanofibers could be prepared at low surface tension and high spinning voltage. Zheng et al. [26] found that compared to the multi-needle electrospinning system, the multi-hole spinneret produced finer fiber diameters and higher yield; this was because the multi-hole spinneret had a more uniform and stronger electric field, except for the area very close to the spinneret. Jian et al. [19] designed a mushroom-shaped spinneret, which could promote the charge accumulation density of a spinning solution, thus reducing the spinning voltage and increasing the fiber yield. According to Refs. [27,28], with the increase in voltage, the charge density on the jet surface increases, resulting in an increase in jet velocity and a decrease in fiber diameter. Although there are many studies on the distribution of electric field intensity, there are few studies on the effect of spinnerets with different electric field distributions on the charge density distribution of jets under the same voltage, which is basically a theoretical derivation.

In our previous study [15], four FSE devices with different spinnerets were designed and compared, namely the modified bubble electrospinning device, the modified FSE device, the oblique section FSE device, and the spherical section FSE (SSFSE) device. It was found that the SSFSE device had the most uniform electric field distribution and higher electric field intensity and could achieve the best nanofiber yield and quality. Moreover, by controlling the uniformity of the electric field intensity, the motion velocity of each jet could be controlled to ensure the uniformity of the fibers. Subsequently, the SSFSE device was further optimized to produce higher-yield and higher-quality nanofibers [21]. Therefore, the influences of spinnerets with different sphere radii on their electric field distribution, jet generation, and nanofiber yield were studied under different spinning voltages. The results indicated that when the sphere radius of the spinneret was 75 mm and the spinning voltage was 40 kV, the SSFSE device could achieve the highest yield and quality of nanofibers. Moreover, the SSFSE device has been used by our group to prepare various functional nanofibers with different structures in batches, which have been applied in fields such as food packaging [2], wound dressings [6], and frictional electric nanogenerators [16]. However, the above studies are all based on experimental research or electric field simulation, and there is a lack of studies on the formation mechanism and mechanical behavior of multiple jets during the SSFSE process, which is not conducive to guiding future practical production.

Herein, a force analysis was conducted on the formation and motion process of multiple jets in the SSFSE process, and the relationship between the jet initial velocity and the electric field intensity was established. The computational fluid dynamics method was used for numerical simulation to analyze the position and velocity distribution of the jets generated in the SSFSE processes using spinnerets with different sphere radii. Finally, the numerical simulation results were verified by experiments using a polymeric solution with low conductivity.

## 2. Spinning Mechanism of SSFSE

**Figure 1:**
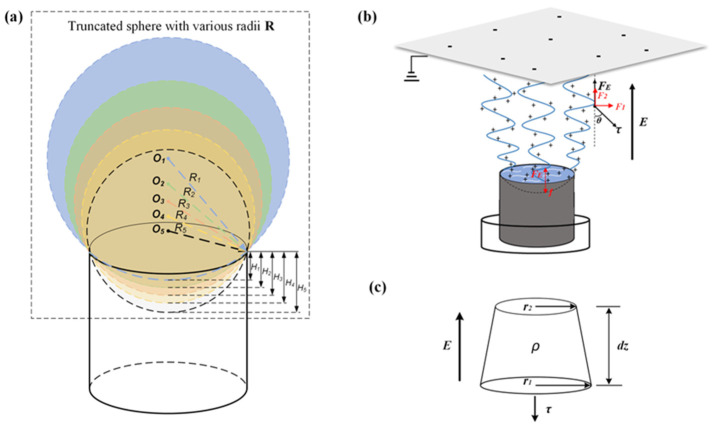
Diagram of the spinneret truncated by various sphere radii (**a**), force analysis of jet (**b**), and jet micro-element (**c**).

The replaceable spinneret of the SSFSE device was a type of copper cylinder with a height of 40 mm and a diameter of 50 mm. As displayed in Figure 1a, these spinnerets were truncated by distinct spheres with corresponding radii of 45 mm, 55 mm, 65 mm, 75 mm, and 85 mm, respectively. When a high electric field was applied to the SSFSE device, the electrohydrodynamic instability induced the free charges in the solution and distributed them at the spinning solution surface, as shown in Figure 1b. The uneven distribution of charges would disturb the steady state of the spinning solution surface, causing it to produce tiny fluctuations. As the electric field intensity continued to increase, multiple peaks would be generated simultaneously at the small fluctuations, where each peak was equivalent to a Taylor cone [25]. The charges were concentrated at the point with the maximum curvature radius of the Taylor cone, resulting in stronger electrohydrodynamic effects. Therefore, when the jet was produced in the SSFSE process, the initial velocity of unit charge movement in the jet could be expressed according to the force acquired at the time. When the voltage is high enough, the electric field force ($F_{E}$) generated is sufficient to overcome the surface tension (γ) of the solution (i.e., $F_{E}>\gamma$), and multiple jets can be generated from the spinning solution surface. Thus, during the excitation of the spinning solution surface, the resultant force exerted on the jet micro-elements can be expressed as follows:

(1) $$m\frac{d^{2}r}{{dt}^{2}}=F_{E}-\gamma$$

(2) $$F_{E}=qE$$

where q is the charge of the jet micro-element, which is mainly determined by the properties of the spinning solution, generally an integer multiple of the elementary charge (1.60 × 10^−19^ C). γ is the surface tension (N/m), and *E* is the electric field intensity (V/m).

According to Newton’s second law, when a jet is pulled up from the solution surface, the resultant force exerted on the jet can also be expressed as F=ma. As shown in Figure 1c, the jet is divided into multiple micro-elements (dz) for further analysis. Considering that the jet radius (r) of the micro-elements remains unchanged ($r=r_{1}=r_{2}$), the mass ($m_{i}$) and acceleration ($a_{i}$) of the *i-th* segment of the micro-elements in the jet can be expressed as follows:

(3) $$m_{i}=\rho \pi r^{2}dz$$

(4) $$a_{i}=\frac{v^{2}}{2dz}$$

Therefore, the instantaneous velocity (v) of the micro-element ejecting from the solution surface can be obtained by substituting Equations (3) and (4):

(5) $$q_{i}E-\gamma =ma_{i}=\frac{\rho \pi r^{2}v^{2}dz}{2dz}=\frac{\rho \pi r^{2}v^{2}}{2}$$

(6) $$v={\left(\frac{2q_{i}E-\gamma }{\rho \pi r^{2}}\right)}^{\frac{1}{2}}$$

where $q_{i}$ is the amount of charge carried by the *i-th* segment jet micro-element, and ρ is the fluid density.

After the jet is pulled from the solution surface, it begins at the stage of unstable motion under the combined action of electric field force, gravity, air resistance, and viscous force (τ) [29,30]. Nonetheless, due to the tiny mass of a single jet in the unstable motion state, environmental factors such as gravity and air resistance can be ignored during unstable motion. Consequently, the viscosity of the spinning solution plays a key role in the movement of the jet, which indicates that the higher the viscosity of the spinning solution, the greater the viscous force on the jet, and the greater the electric field force required to propel the jet towards the collector direction. From the above, the effects on the unstable motion of the jet can be categorized as the combined action of the electric field force and the viscous force ($\tau =av+bv^{2}$, where a and b are constants determined by the viscosity of the solution). As depicted in Figure 1b, the vertical upward direction of the electric field force can cause the upward movement of the jet. The direction of the viscous force is opposite to the direction of the jet movement, which is the friction that impedes the relative deformation trend of the fluid and can be decomposed into the horizontal and vertical forces, respectively. Therefore, the resultant force ($F_{1}$) of the jet in the horizontal direction is to restrain the unsteady bending and swinging of the jet, while the resultant force ($F_{2}$) of the jet in the vertical direction can cause the continuous movement of the jet towards the collector and the formation of nanofibers by stretching and thinning, which can be expressed as follows:

(7) $$F_{1}=\tau \sin\theta$$

(8) $$F_{2}=qE-\tau \cos\theta$$

Thus, according to the model of jet motion and the force analysis of the jet motion process, it can be concluded that the electric field intensity has a significant impact on the formation and motion of the jet in the SSFSE process. Therefore, the numerical simulation can further comprehend its spinning mechanism and aid in the fabrication of nanofibers with more uniform diameter distribution.

## 3. Model

When the jet is ejected from the spinning solution surface, only the upward movement process driven by the electric field is considered. During the movement process of the jets, the Maxwell equations, the mass conservation equation, the charge conservation equation, the momentum conservation equation, and the energy conservation equation are involved [31]:

(1) Maxwell equation

(9) $$\frac{\partial q}{\partial t}+\nabla \cdot \vec{J}=0$$

(10) $$\nabla \times \vec{E}+\frac{1}{c}\frac{\partial \vec{B}}{\partial t}=0$$

(11) $$\nabla \times \vec{H}-\frac{1}{c}\frac{\partial \vec{D}}{\partial t}=\frac{1}{c}\vec{J}$$

where t is the time, $\vec{E}$ is the electric field, $\vec{J}$ is the current, $\vec{B}$ is magnetic induction, $\vec{H}$ is the magnetic field, and c is the velocity of light in a vacuum.

(2) Conservation of mass

(12) $$\frac{D\rho }{Dt}=\frac{\partial \rho }{\partial t}+\nabla \cdot \left(\rho \vec{v}\right)=0$$

where ρ is the density of the jet, and $\vec{v}$ is the velocity of the jet.

(3) Momentum equation

(13) $$\rho \frac{D\vec{v}}{Dt}=\nabla \cdot \vec{t}+\rho \vec{f}+q\vec{E}+\left(\nabla \vec{E}\right)\cdot \vec{P}$$

where $\vec{t}$ is the stress tensor, $\vec{f}$ is the body force, and $\vec{P}$ is the polarization.

(4) Energy equation

(14) $$\rho c_{p}\frac{DT}{Dt}=Q_{h}+\nabla \cdot \vec{q}+\left(\vec{J}-q\vec{v}\right)\cdot \left(\vec{E}+\frac{1}{c}\vec{v}\times \vec{B}\right)\;-\left(\vec{E}+\frac{1}{c}\vec{v}\times \vec{B}\right)\cdot \frac{D\vec{P}}{Dt}-\left(\vec{M}+\frac{1}{c}\vec{v}\times \vec{P}\right)\cdot \frac{D\vec{B}}{Dt}+Q_{f}$$

where $c_{p}$ is specific heat capacity at constant pressure, *Q_h_* is the source term, $\vec{q}$ is the heat, $\vec{M}$ is the magnetization, and *Q_f_* is the energy loss caused by the air drag.

It is assumed that a jet is an incompressible fluid and that its density (ρ) in the whole process of motion is constant. As a result, three models are primarily utilized to simulate the motion trajectory of polymer jets; these are introduced as follows:

(1) Magnetohydrodynamics (MHD) model

MHD coupling is used to simulate the interaction between the flow field and the electric field by introducing the Lorentz force ($\vec{F}$) and Joule heating ($J_{h}$) as additional source terms in the fluid momentum equation and energy equation [32,33], respectively, which can be derived as follows:

(15) $$\vec{F}=\vec{J}\times \vec{B}$$

(16) $$J_{h}=\frac{1}{\sigma }\vec{J}\cdot \vec{J}$$

where σ is the electric conductivity of the media.

Therefore, the electric field ($\vec{E}$) can be expressed as follows:

(17) $$\vec{E}=-\nabla \varphi -\frac{\partial \vec{A}}{\partial t}$$

where φ is the scalar potential, and $\vec{A}$ is the magnetic vector potential.

(2) Turbulence model

The Reynolds number ($R_{e}$) of fluid motion is calculated to determine the fluid flow state. In the process of SSFSE, the jet bends under the action of the viscous force. According to Equation (18) [34], $R_{e}=2471.6>2000$. Thus, the renormalization group (RNG) *k-*ε turbulence model is selected to describe the turbulent flow in the SSFSE process, and the velocity and length of the fluid motion can be solved.

(18) $$R_{e}=\frac{\rho vL}{\mu }$$

where ρ is the fluid density, v is the jet velocity, L is the characteristic length (diameter of the spinneret), and μ is the dynamic viscosity of the fluid.

(3) Volume of fluid (VOF)

VOF is a Euler method used to solve the state of free fluid when the jet reaches the mesh cell of the computational domain (air). In the initial state, no polymer fluid is solved in the cell of the computational domain, and the phase fraction of the polymer fluid $\alpha_{q}$ is equal to 0. When a part of fluid exists in the cell, the phase fraction is $0<\alpha_{q}<1$; when the cell is filled with fluid, $\alpha_{q}=1$. Therefore, Equation (19) shown below is used to solve the phase fraction [32,35]:

(19) $$\frac{\partial \alpha_{q}}{\partial t}+\nabla \cdot \left(\alpha_{q}\vec{v}\right)+\nabla \cdot \left[\alpha_{q}\vec{v_{r}}\left(1-\alpha_{q}\right)\right]=0$$

where $\vec{v_{r}}=\vec{v_{l}}-\vec{v_{g}}$ is the vector of relative velocity, and $\vec{v_{l}}$ and $\vec{v_{g}}$ are the velocities of the polymer fluid and air, respectively.

## 4. Numerical Simulation of Jet Motion Process

The CFD simulation results are determined by different solution properties and spinning parameters, which can be used to optimize and control the jet motion in the electrospinning process. However, because of the rapid fluctuations in jet motion during the SSFSE process, accurately measuring jet velocity in practical testing is challenging. Hence, the processes of the SSFSE device with various sphere radii were numerically simulated; this was also utilized to analyze the jet motion trajectory and velocity changes during movement, revealing the motion law of the jet.

### 4.1. Computational Domain

As shown in the schematic diagram of the SSFSE device (Figure 1b), the model of the jet motion process can be regarded as a rotationally symmetric structure. This allows it to be described using a straightforward 2D axisymmetric geometry, which saves computing resources during the real operation. The computational domain was set to a radius of 100 mm (0 ≤ x ≤ 100 mm), and the distance between the spinneret and the collector was 180 mm (0 ≤ y ≤ 180 mm). Since the jet motion could be distributed at any position in the SSFSE process, a dense quadrilateral element was applied as the surface mesh. The 2D mesh was composed of 449,375 quadrilateral elements and 450,776 nodes. The average orthogonal quality of the mesh was close to 1, and the average inclination was 1.306 × 10^−10^. The properties of the model materials are displayed in Table 1.

### 4.2. Boundary Conditions

The computational domain exhibited three boundaries, and each boundary condition was set as follows:

(1) Velocity inlet

**Figure 2:**
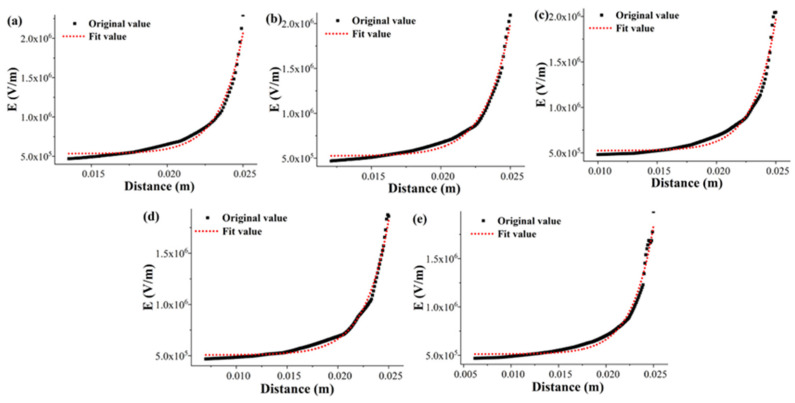
Simulated electric field intensity value and fitting curve of the spinneret with different sphere radii: (**a**) 45 mm, (**b**) 55 mm, (**c**) 65 mm, (**d**) 75 mm, (**e**) 85 mm.

As the structure of the spinneret is a rotationally symmetric geometry, the simulated radial electric field intensity generated on the solution surface varies with the position, resulting in different initial jet velocities at the different positions of the inlet boundary. Therefore, to continuously set the initial velocity of each jet generated on the solution surface, the electric field intensity values from the radial center of the spinneret to its edge were fitted and substituted into Equation (6) to obtain the initial velocity of the jet. Therefore, based on the electric field simulation results of the SSFSE device with the corresponding sphere radius at 40 kV in our previous study [21], the electric field intensity values on the solution surface at the inlet boundary, i.e., from the radial center of the spinneret to its edge, were obtained. Based on the original values of these electric field intensities, an exponential function (Equation (20)) with higher correlation (R^2^) was used for fitting, and the results are shown in Figure 2 and Table 2.

(20) $$E=Ae^{\frac{x}{t}}+y_{0}$$

**Figure 3:**
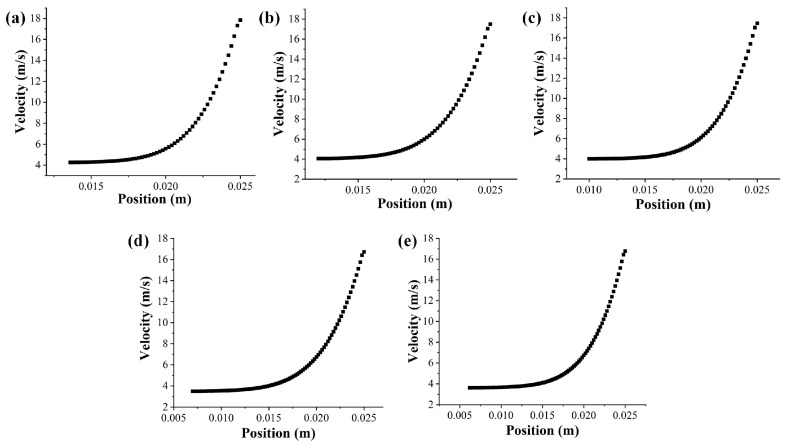
The initial velocity changes with the inlet position of the spinneret with different sphere radii: (**a**) 45 mm, (**b**) 55 mm, (**c**) 65 mm, (**d**) 75 mm, (**e**) 85 mm.

Meanwhile, on the basis of our previous work [21], it was found that the minimum required electric field intensity was about 468,463 V/m according to the position of the jet generation, and the charge of the jet was about 7.7478 × 10^−8^ C. The charge of the jet should be an integer multiple of the elementary charge; so, the charge of the jet was set to 8.05 × 10^−8^ C. Therefore, at the inlet boundary, the specific location of the jet generated on the solution surface (∆x) could also be obtained by calculation, as shown in Table 2. The fitting model of the electric field intensity was substituted into Equation (6), assuming that the initial radius of the jet was 500 μm, and the model of the initial velocity changing with the inlet position could be obtained, as shown in Figure 3.

The potential value at the inlet was set to 40 kV, and the volume fraction of phase 2 was set to 1. The rest of the computational domain was initialized as air.

(2) Free surface: zero normal gradient for all variables, ∂∅/∂n=0.

(3) No-slip boundary: zero velocity relative to the wall, $u_{wall}=0$.

### 4.3. Other Parameters

The jet was assumed to be an incompressible fluid, and the operating pressure was set to 101,325 Pa. Due to the very small mass of the jet, the effect of gravitational acceleration on the jet was ignored. In the simulation, the finite difference interpolation scheme and explicit method were used to solve these volume fraction equations. The face values of the volume fraction were interpolated by using a geometric reconstruction scheme. The Runge–Kutta method was used for solving, with a solution step of 0.001 s, and to ensure fast convergence. Moreover, the monitoring window was set to record data every 50 iterations.

### 4.4. Results and Discussion

#### 4.4.1. Numerical Simulation Results

**Figure 4:**
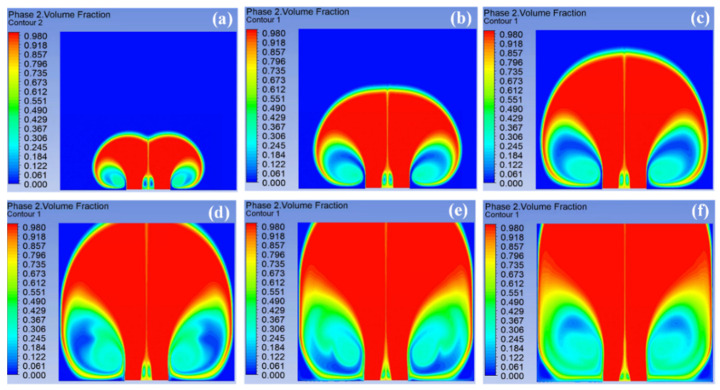
The change in the volume fraction of the multiple jets at 0.03 s (**a**), 0.06 s (**b**), 0.09 s (**c**), 0.12 s (**d**), 0.15 s (**e**), and 0.18 s (**f**).

Figure 4 exhibits the volume fraction distribution of multiple jets at various time snapshots during the spinning process of a spinneret with a sphere radius of 75 mm, which showed that the jets could fully gather at the entrance at 0.03 s. Over time, the polymer solution steadily approached the collector. Due to the high velocity of the fluid, it could reach the collector completely at 0.15 s. In addition, the divided mesh was relatively dense, so that jets could be generated at every position of the entrance in an ideal case. The SSFSE device could generate many jets simultaneously, and multiple jets moved together, which allowed most of the jets to be distributed in the entire computational domain after 0.15 s.

**Figure 5:**
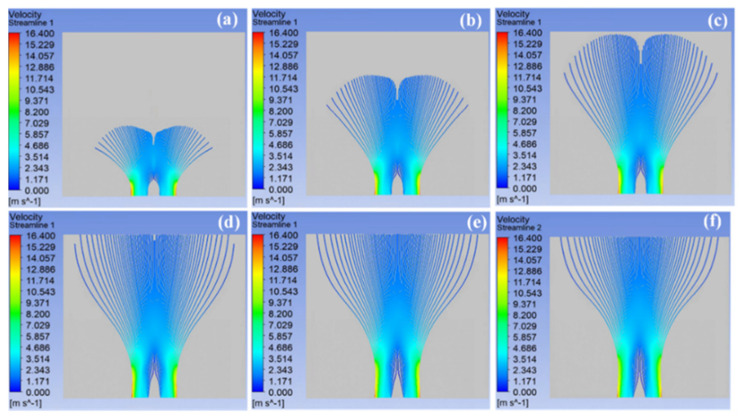
The change in the velocity streamline of the multiple jets at 0.03 s (**a**), 0.06 s (**b**), 0.09 s (**c**), 0.12 s (**d**), 0.15 s (**e**), and 0.18 s (**f**).

Figure 5 shows the velocity of multiple jets generated by the spinneret with a sphere radius of 75 mm at different time snapshots, which represent the motion trajectory and velocity distribution of the multiple jets. The jet velocity at the edge of the spinneret was always the highest, which was related to the highest electric field intensity at this location. The initial stage of the jet movement was the fastest and the most stable, and when the fluid moved at high velocity, it did not produce large bending disturbance under the action of inertia force. In the SSFSE process, the viscous force of the jet during motion remained unchanged with the unchanged solution density. However, as the distance from the collector to the jet decreased, the electric field intensity and charge of the jet gradually diminished, which resulted in a smaller electric field force for the jet, a gradual decrease in jet velocity, and the unstable motion of the jet. The unstable motion of the jet would cause it to gradually spread to both sides. Consequently, although the jet velocity at the edge of the spinneret was the largest, the outward expansion phenomenon was more obvious, because of its excessive velocity and unstable movement, resulting in the latest time of its arrival at the collector.

**Figure 6:**
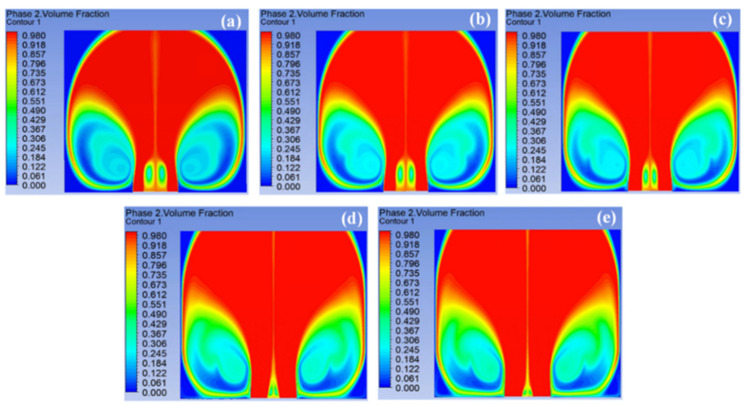
The volume fraction of spinneret with different sphere radii at 0.15 s: (**a**) 45 mm, (**b**) 55 mm, (**c**) 65 mm, (**d**) 75 mm, (**e**) 85 mm.

Figure 6 shows the volume fraction of the multiple jets generated by the spinnerets with different sphere radii at 0.15 s. It was found that with the increase in the sphere radius, the range of multiple jets generated on the solution surface became larger, so that the number of multiple jets reaching the collector also increased, which was related to the distribution of the electric field intensity. These simulation results were consistent with the experimental data in a previous work [21]. The more jets reaching the collector, the higher the fiber yield obtained.

**Figure 7:**
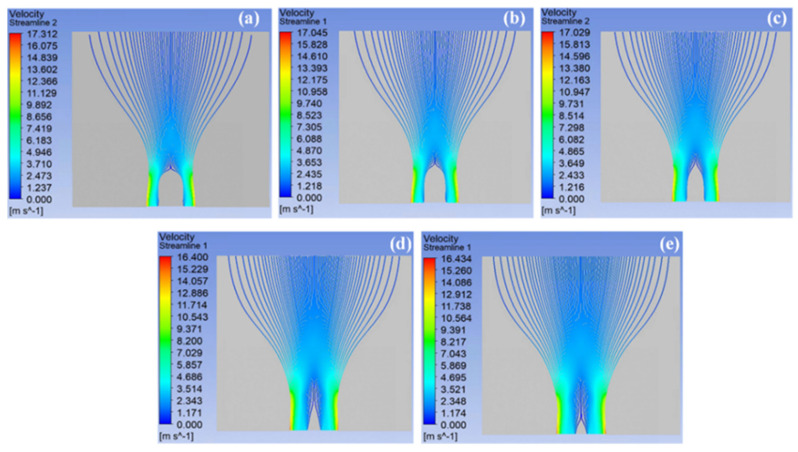
The velocity streamlines of spinneret with different sphere radii at 0.15 s: (**a**) 45 mm, (**b**) 55 mm, (**c**) 65 mm, (**d**) 75 mm, (**e**) 85 mm.

Figure 7 shows the velocity of multiple jets generated by the spinnerets with different sphere radii at 0.15 s. Compared with other spinnerets, the jet velocity at the edge of the spinneret with a sphere radius of 45 mm was the largest, at 17.312 m/s. With the increase in the sphere radius, the maximum jet velocity at the edge of the spinneret gradually decreased. The main reason was that the tip discharge phenomenon of the spinneret was gradually weakened, and the electric field intensity decreased. According to the law of mass conservation ($Q=\pi r^{2}v$), the larger the jet velocity, the smaller the fiber diameter obtained. At the same time, with the increase in the sphere radius, the maximum velocity of the jet at the spinneret edge remained at a long distance, which was related to the uniform electric field distribution in the SSFSE process. This increased the duration of the stable motion stage of the jet and enabled a uniform fiber diameter distribution. In addition, the increase in the sphere radius made the outward expansion of the jet during unstable motion more obvious.

**Figure 8:**
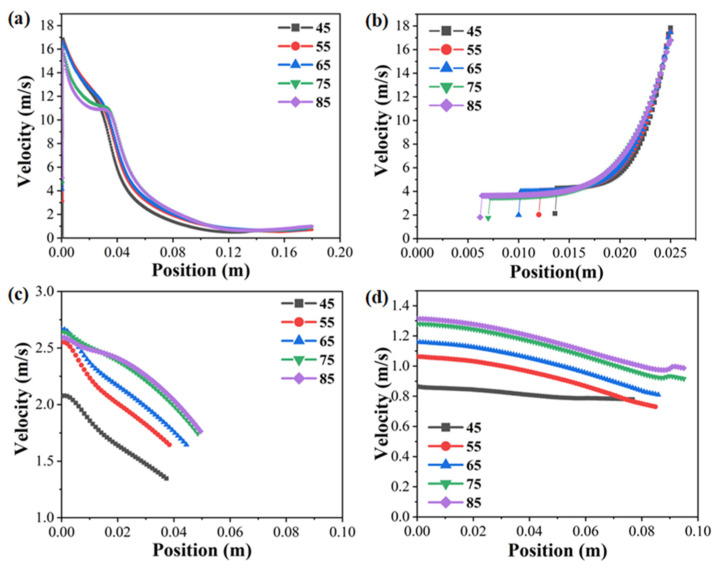
The velocity distribution of a single jet at the edge of the spinneret from the solution surface to the collector (**a**) and the velocity distribution of multiple jets at 0 mm (**b**), 90 mm (**c**), and 180 mm (**d**) from the solution surface at 0.15 s.

To directly compare the jet velocity distribution of spinnerets with various sphere radii, the velocity distribution curves of a single jet at the edge of the spinneret from the solution surface to the collector (Figure 8a), as well as the velocity distribution curves of multiple jets at distances of 0 mm (Figure 8b), 90 mm (Figure 8c), and 180 mm (Figure 8d) from the spinning solution surface at 0.15 s, are shown in Figure 8. As shown in Figure 8a, after the single jet at the edge of the spinneret was generated from the solution surface, the distance between the jet and the solution surface gradually increased, and the axial electric field intensity of the interval where the jet was located decreased accordingly with the upward movement of the jet, resulting in a gradual reduction in the upward electric field force on the jet and its velocity. In addition, the surface electric field intensity of the spinneret decreased with the decrease in its sphere radius, and the initial velocity of the jet also decreased. Therefore, in the initial motion stage (about 0–40 mm from the solution surface), the corresponding velocity of the single jet at the edge of the spinneret decreased with the increase in its sphere radius.

It can be seen in Figure 8b that with the increase in the sphere radius, the range of jets generated on the solution surface increased, and the average initial velocity of the jet decreased accordingly. However, as the distance between the jet and the solution surface continued to increase, the axial electric field intensity of the spinneret with a larger sphere radius was higher, resulting in a greater upward electric field force on the jet and a slower decrease in jet velocity. As a result, in the subsequent movement stage (about 40–120 mm from the solution surface), the jet generated by the spinneret with a large sphere radius had a higher velocity, as shown in Figure 8c. At the last stage of the movement of a single jet at the edge of all the spinnerets (about 120–180 mm away from the solution surface), it could be seen that the jet was hardly affected by the electric field force, and its velocity tended to be constant (Figure 8a). Moreover, the jet generated by the spinneret with a large sphere radius still had a higher velocity, which was further confirmed by Figure 8d. In addition, according to the velocity distribution of multiple jets at the distance of 90 mm and 180 mm from the solution surface, due to the current instability of jet motion, the jet trajectory seriously expanded outward, resulting in lower velocities of jets that were further away from the axis in the radial direction. Therefore, due to the decrease in the stretching ratio caused by the reduction in jet velocity, the diameter distribution of the nanofibers electrospun by the spinneret with a larger spherical radius was more uniform, but the diameter was larger, which was validated by the fiber diameter distribution measured through experimental studies in previous works [21].

#### 4.4.2. Experimental Verification

Through experimental research, the accuracy and wide applicability of the numerical simulation method can be further verified. A spinning solution with low conductivity has a relatively constant jet diameter before its jet begins to move unstably [36], which is more conducive to the capture of jet motion and the calculation of velocity. Therefore, a low-conductivity spinning solution was used for experimental verification, and its jet velocity during the stable stage was analyzed by a high-speed camera to verify the numerical simulation results.

**Figure 9:**
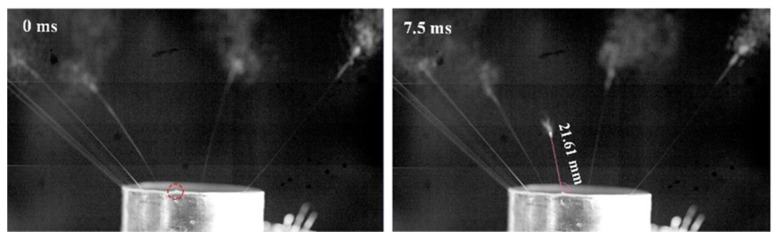
The change in jet motion position with time. The red circle presents the position where a single jet is pulled from the spinning solution surface to measure its velocity.

Thermoplastic polyurethane (TPU, 9380A) particles were dissolved in *N*, *N*-Dimethylformamide/acetone (*w*/*w* = 70/30) to prepare a spinning solution with a concentration of 15 wt%. The properties of the spinning solution were measured and included a density of 854.73 kg/m^3^, a conductivity of 19.1 μS/cm, a surface tension of 32.15 mN/m, and a viscosity of 254.3 mPa·s. The spinning voltage was set as 50 kV, and the distance between the spinneret and collector was set as 180 mm. The preparation process of TPU nanofibers using the SSFSE device with a sphere radius of 75 mm was studied by a high-speed camera (VRI-phantom-VEO-410L, AMETEK, Inc., Berwyn, IL, USA, 4000 fps). The relationship between the moving distance and the time of the TPU jet was analyzed and was used to calculate the actual moving velocity of the TPU jet to validate the simulation results. Based on the images captured by the high-speed camera, instantaneous images were selected at 0 ms (the single jet was pulled from the spinning solution surface) and 7.5 ms, as shown in Figure 9. The length of stable linear jet segment from 0 ms to 7.5 ms was 21.61 mm, and the average velocity of the jet in this segment could be calculated as 2.88 m/s.

**Figure 10:**
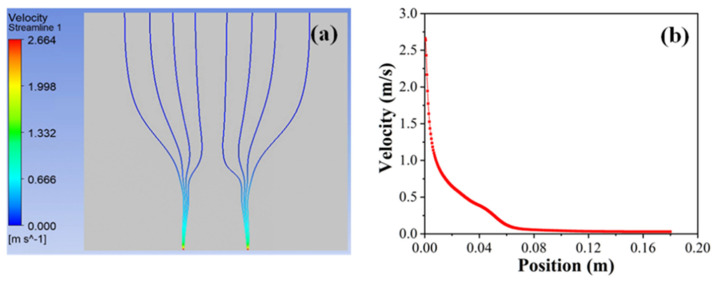
The motion process (**a**) and the velocity change at the edge of spinneret (**b**) of TPU jets.

Based on the above experimental parameters, the numerical simulation of the jet motion in the SSFES process was carried out. According to the experiment results, the charge of the jet was set as 9.66 × 10^−9^ C. In addition, it was observed that the jets were generated only at the edge of the spinneret; so, it was assumed that the jets were generated only at the solution surface within 1 mm from the spinneret edge. According to the above parameters, $R_{e}$ calculated using Equation (18) was 484.3. Therefore, a laminar flow model was used to simulate the TPU jet motion, and the other simulation settings were the same as those above. As displayed in Figure 10, the maximum velocity of the TPU jet at the solution surface was 2.66 m/s, which was much closer to the above experimental result (2.88 m/s), with an accuracy of nearly 92.3%.

## 5. Conclusions

In this paper, the formation mechanism and motion process of jets on the free solution surface during the spinning processes of SSFSE devices with different sphere radii were studied. The key role of the electric field force in the formation and motion of the jets was clarified, and a relationship between the initial jet velocity and the electric field intensity distribution on the solution surface was established. Then, to numerically simulate the motion velocity and trajectory of multiple jets under different electric field intensity distributions, the MHD model was introduced, and the turbulence model and VOF model were combined to simulate the motion trajectory of multiple jets entering the solution zone. The results showed that as the distance between the multiple jets and the collector continuously decreased, the decrease in electric field intensity weakened the electric field force on the jets. The velocity of the jets gradually decreased, and the jets began to enter unstable motion, causing them to gradually spread to both sides.

Meanwhile, as the sphere radius increased, the area where multiple jets were generated increased, and the interaction force between the jets increased, resulting in a more pronounced outward expansion of jet trajectory. In addition, the increase in the sphere radius led to a less obvious decrease in jet velocity, leading to a larger fiber diameter and a more uniform diameter distribution. Taking into account various factors, the optimal spinneret for SSFSE was determined to have a sphere radius of 75 mm. Finally, experiments were conducted using a low-conductivity TPU spinning solution to validate the proposed model and numerical simulation method. It was found that the numerical results were consistent with the experimental results, indicating the applicability of the developed SSFSE device and the proposed numerical method. Accordingly, this work can play a guiding role in effectively enhancing the jet number per unit area of solution surface in practical production, thereby realizing continuous, well-distributed, and efficient preparation of micro-/nanofibers.

## Figures and Tables

**Table 1:** The properties of model materials.

Materials	Viscosity (mPa.s)	Conductivity (S/m)	Surface Tension (mN/m)	Density (kg/m^3^)
Air	2 × 10^−3^	1.0 × 10^−10^	-	1.25
PAN solution	334	2.37 × 10^−2^	36.295	953.7

**Table 2:** The electric field fitting model of the spinneret with different sphere radii.

R	E	R^2^	∆x (mm)
45	0.163 × exp(x/0.00156) + 534,291.563	0.978	13.5
55	1.540 × exp(x/0.00182) + 525,994.943	0.986	12.1
65	2.550 × exp(x/0.00189) + 524,320.590	0.977	10
75	31.178 × exp(x/0.00235) + 506,983.252	0.988	7
85	25.980 × exp(x/0.00231) + 511,653.892	0.980	6.2

## Data Availability

The original contributions presented in this study are included in the article. Further inquiries can be directed to the corresponding author.
